# Humans and Goats: Improving Knowledge for a Better Relationship

**DOI:** 10.3390/ani12060774

**Published:** 2022-03-18

**Authors:** Stefania Celozzi, Monica Battini, Emanuela Prato-Previde, Silvana Mattiello

**Affiliations:** 1 Department of Agricultural and Environmental Sciences—Production, Landscape, Agroenergy, University of Milan, 20133 Milan, Italy; stefania.celozzi@unimi.it (S.C.); silvana.mattiello@unimi.it (S.M.); 2 Department of Pathophysiology and Transplantation, University of Milan, 20133 Milan, Italy; emanuela.pratoprevide@unimi.it

**Keywords:** *Capra aegagrus hircus*, animal welfare, human–animal relationship, interspecific interactions, attitude, empathy, behaviour, communication, stockperson

## Abstract

**Simple Summary:**

A good relationship between humans (e.g., farmers, owners) and farm animals is vital for the well-being of both parties: on the one hand, people are satisfied with their work, which becomes less stressful and more profitable, and may receive social benefits in terms of education or animal-assisted therapy; on the other hand, animals are rewarded by the presence of humans and are not afraid of them. Goats have high cognitive and communicative abilities towards humans: recognising these abilities helps humans to work properly on the quality of this relationship that is built from the first hours of the goat kids’ life, thanks to frequent and positive contacts (e.g., stroking, talking in a calm voice). Improving the quality of this relationship is an investment in the future of livestock farming and meets public demands for ethical and sustainable production. This review outlines the characteristics and predisposing factors for the establishment of a good human–goat relationship and for its evaluation.

**Abstract:**

There is consensus that the quality of the human–animal relationship (HAR) is relevant to guarantee appropriate levels of animal welfare. Given the impact that HAR may have on both goats and human beings, the aim of the present review is to elucidate: (1) how humans and goats communicate; (2) which are the factors affecting human–goat interactions; (3) how we can measure the quality of this relationship. The systematic review led to the selection of 58 relevant articles. Effective human–goat communication takes place by means of visual, tactile and auditory stimuli and, to a less extent, via olfactory and gustative stimuli. Goats have well-developed socio-cognitive abilities and rely on humans to get relevant information. A deep knowledge of goats’ communication means and socio-cognitive abilities may greatly help improving the human–goat relationship. Management practices (e.g., rearing methods, amount and quality of interactions), as well as genetic selection for suitable individual traits, may contribute to improving HAR. Several measures to assess the quality of HAR have been validated, including avoidance in the pen and at the feeding rack and latency to first contact. Finally, farmers’ attitudes and empathy with goats, as well as their motivation to work with animals, should be improved through appropriate training.

## 1. Introduction

The relationship between humans and goats has been dramatically changing during the last millennia, evolving from bare hunting for subsistence to intensive farming. This occurred through a long domestication process, initiated about 10,000 years ago, which led the still existing wild ancestor (*Capra aegagrus*) to adapt to farming conditions and to a close relationship with humans. Domestic goats (*Capra aegagrus hircus*) are now a widely used and increasingly economically important commercial species, reared in a variety of systems, going from very intensive systems to extensive pasture-based systems. Small holdings and family farms are also widespread all around the world, especially in rural communities in the mountain or in tropical and subtropical areas. Goat population numbers have been increasing in the last 30 years, doubling their consistence and reaching a total population of 1.09 milliard heads, most of which are located in Asia (51%) and Africa (43%) (data retrieved from FAOSTAT: https://www.fao.org/faostat/en/#data/QCL; last access on 20 January 2022). Therefore, goat farming plays an important role in the economy of many countries, and it deserves attention in order to make it efficient and profitable, as well as for ethical reasons.

Improving the human–animal relationship may represent an effective low-cost strategy to increase animal performances and welfare, with no need of high investments, and might therefore be implemented even in low-income countries, where goats are widespread. For this aim, a very good knowledge of the mechanisms underlying the formation and consolidation of this relationship is crucial.

Multiple interactions between individuals lay the foundations for a consolidated relationship between the parties: the predictable expectations determined by these interactions influence the nature and perception of future encounters [1]. Bonds can be formed between animals of the same or different species, but this review will only focus on the relationships that develop between humans and animals, particularly between humans and goats. The relationship resulting from progressive interactions between humans and animals is a dynamic process usually called the “Human–Animal Relationship” (HAR) [2] and is also defined as “the degree of relatedness or distance between the animal and the human” [3]. This relationship implies that animals are able to memorise interactions with humans: the recall of the type of interaction has a strong and long-lasting impact on animal welfare [1]. Thus, the establishment of this relationship implies a mutual recognition. However, many animal species can both generalise [4] and discriminate in their experience and recognition of humans (cows: [5], sheep: [6], goats: [7]). This ability is also true for the stockperson who can establish an individual relationship with a few animals and a general HAR with the herd [8]. According to [9], humans may be perceived by farm animals as predator, prey, part of the environment without social significance, symbiont and conspecific, even if these roles are not mutually exclusive and sometimes questionable (e.g., symbiont and conspecific) [3]. Furthermore, three categories of emotions can describe the impact of humans on animals: negative, neutral, positive emotions. Despite centuries of domestication, it is likely to assume that prey species, such as goats, may perceive humans as predators to be feared, although close and frequent contacts may change this perception [10].

Recently, in [11] updated the Five Domains Model modifying the name of the fourth Domain from Behaviour to Behavioural Interactions. The revision of this Domain reflects the conscious behavioural choices the animals make and their perceptions of external circumstances towards humans. Animals actively seek for contacts with people and positive interactions are rewarding for them, increasing the motivation to engage with humans. Hence, the improvement of HAR quality is an opportunity to enhance both welfare and affective states. The authors list some behaviours of humans in specific situations that can positively influence the animals’ affective state, e.g., humans offering foods, the presence of humans providing feelings of safety, humans participating in enjoyable routine activities or calming animals in threatening circumstances [11]. In contrast, if the behaviour of humans prevents the animals from receiving such a pleasant reward (e.g., limited human contacts and/or actions directly unpleasant, association of the human presence during threatening circumstances), animals may experience negative affective states. 

On the farm, the quality of the stockmanship has the greatest potential to determine the quality of HAR. The improvement of the relationship between stockpersons and goats leads to a number of benefits for both parties; conversely, a poor relationship leads to clear impairments. From the farmer’s point of view, the quality of HAR influences the performance of animals and the ease of handling them, as well as the satisfaction for the work [12]. It is well known that poor HAR negatively affects the productivity of cows [13,14], pigs [15] and poultry [16]. To the best of our knowledge, only one study investigated the effect of HAR on goat milk production [17], showing an impaired ejection of alveolar milk in goats that had received few contacts with humans. The results of this study highlight how fear of humans negatively affects the productivity of goats, as fostered by [18]. However, the quality of HAR did not affect goat milk composition (e.g., content of fat or protein; [19]), probably due the chemical stability of goat milk that is only altered during diseases (e.g., [20,21]). Considering other studies on the effects of HAR on goats’ productive performance, heavier weight gains were recorded in response to a greater possibility of contact with humans in feral goats kept in captivity [22]. In [19] also observed a significant increase in heart girth, possibly due to changes in appetite or nutrient uptake and energy expenditure in goats subjected to positive physical manipulation. Furthermore, in [23] showed that adverse manipulation might have negative consequences on placental morphology and foetal survival in pregnant goats. Interestingly, the same study also showed that human positive interactions with pregnant goats lead to a positive effect on maternal care and on the behaviour of their goat kids. 

A relevant side effect of investing in the formation of a positive HAR concerns the consumer’s perception of the sector [24]: high standards of animal welfare are an important societal issue, and there is the expectation that animals raised in production industries receive good care and are treated humanely [25]. The failure to meet these expectations diminishes public acceptance and trust, posing a threat to the “social licence” to farm (i.e., public acceptance and support), and in turn, leads the sector to economic losses. Hence, providing care and offering opportunities for the animals to enjoy positive experiences helps meeting consumers’ expectations about the animals’ rights to have a life worth living [26].

From the goat’s point of view, besides being a reward for animals and a social enrichment, good HAR quality can balance the negative experiences the animals usually have in the farm (e.g., vaccinations, mutilations). Early and frequent social interactions with humans can help to reduce stress due to manipulation procedures [27], such as sperm collection by transrectal ultrasound-guided massage of accessory sex glands in goat bucks [28] and the transport handling phase [29]. Habitual interactions with humans can also contribute to the reduction of stress due to adaptation to a new environment such as a laboratory [30] or a new farming system [22].

Although no information is available on goats, a good HAR may positively influence stress resilience in farm animals (heifer: [31]). Positive contacts with humans can affect the physiological response to stress in animals, improving their immune response and thus, their resistance to diseases (poultry: [32]. However, at present there is a lack of studies concerning the effect of human contact on the health of other livestock species, including goats [33]. Alcedo et al. [34] suggested that positive human–animal interactions may be important in promoting goat health through gentle treatment during practices such as deworming or during situations where welfare is poor, such as disease, gestation and kidding, although these aspects were not directly investigated in this study. A good HAR can also have a positive effect on the long-term mood and cognitive abilities of goats, as observed in rescued goats subjected to gentle treatment for two years after having previously experienced a situation of poor welfare [35]. Similarly, in [36] highlighted how the type of experience lived with humans can influence the ability of goats to correctly interpret the visual signals of human beings and consequently goats’ behaviour of choice and approach.

Furthermore, in [37] stressed that stockpeople must work on the quality of the relationship with small ruminants (and thus also with goats), especially when they are not used to frequent handling, such as in the case of extensively reared meat breeds. This is one of the major concerns included by [38] in their evaluation of welfare issues in extensive production systems. Extensively reared livestock are subjected only to sporadic and seasonal encounters with humans, frequently associated to aversive situations (e.g., vaccination, herding for weaning). Based on the experience on cattle and sheep, the authors suggest strategies to mitigate stress and fear reactions in animals, such as the training of young animals to stockpeople moving horseback or using motorcycles, the avoidance of unnecessary force or noise, the provision of food during stressful events [38]. 

A good human–animal relationship can be useful in the scientific field, for example, when it is necessary to make behavioural observations, in order not to influence the results [39]. Cox et al. [40] also stressed the importance of proper care and management of animals in order to obtain valid data in scientific research.

The importance of assessing the quality of HAR is a fundamental part of on-farm welfare assessment protocols. This criterion has been addressed in the welfare assessment protocol for goats reared in intensive conditions developed by the EU-funded project “Animal Welfare indicators” (AWIN) [41]. 

The human–goat relationship can also be seen from a social and cultural point of view, which goes beyond goats’ mere production role. For example, in some African populations, goats are rarely slaughtered to satisfy the need for food, and they are rather kept for other “intangible” reasons, such as building and sustaining reciprocal ties, gaining prestige and respect, or for rituals [42]. In more developed countries, goats can be seen as non-food producing animals also in educational programmes. For example, a good human–goat relationship can have beneficial effects on children’s development and education. Loyd et al. [43] examined the attitudes of parents of middle school children in an urban county in the south-eastern United States towards the use of goats in a farm to school program. The study showed that parents had a positive perception of goats, as they stimulated the interest and enthusiasm of their children for school, increasing their sense of responsibility through the care of the animals and allowing them to spend more time outdoors. Similarly, in [44] highlighted how the experience of daily care of goats in a Tokyo elementary school contributed to a greater familiarity of children with these animals and to stimulate ideas on human–goat coexistence. Moreover, in [45] reported that the use of goats in Japanese schools stimulated children’s interest and empathy towards these animals, an attitude of respect for living beings in general, a greater sense of responsibility and encouragement to learning. The goats also strongly stimulated the sense of collaboration of the children for taking care of these animals. In some cases, therapeutic effects of goats on children were found, such as the reduction of the frequency of problematic behaviours and of the reluctance to go to school. Goats also played a positive role in calming children’s hurt feelings when something unpleasant happened at school. 

In addition to the positive effect that goats had on children, contact with these animals also had positive repercussions for disabled people. In fact, multiply disabled persons established a positive social bond with goats, which was beneficial for increasing patients’ attention, active participation and expression of joy [46].

In conclusion, human–goat interactions may have important impacts on animals for production purposes, and also on human beings for education, recreational and cultural purposes, and for ethical reasons. Therefore, the aim of this review is to present the state of the art of the existing knowledge of the mechanisms underlying the formation and consolidation of this relationship. In particular, we try to elucidate: (1) how humans and goats communicate; (2) which are the factors affecting human–goat interactions/relationship; (3) how we can measure the quality of this relationship. We conclude with recommendations on how to improve the quality of such relationship, based on the results of the previous sections.

## 2. Materials and Methods

The scientific literature on human–goat relationship and communication published up until December 2021 was searched in Web of Science and Scopus electronic Databases. Only full-text articles in the English language (both English and American spelling) were included in the search. The reference population included goats of all ages and sexes, either domestic, feral or wild, under any condition (from intensive farming to free-ranging animals, including also goats in petting zoo and residential institutions for disabled people). Papers were searched in different subject areas concerning Agricultural Sciences, Agriculture Dairy Animal Sciences, Veterinary Sciences, Biological Sciences, Behavioural Sciences, Multidisciplinary Sciences, Ecology, Neurosciences, Zoology, Psychological Sciences, Interdisciplinary Social Sciences and Communication.

The keywords searched were goat$ AND human$ OR farmer$ OR stockperson$ OR stockpeople OR owner*; goat$ AND interaction$ OR communication$ OR relation* OR care. These keywords were combined in different ways. 

This process led to a total number of 66 articles, excluding those which were present in both databases. Fifteen further articles were added via the citation method. The abstracts of 81 articles were subjected to a preliminary screening process and only papers answering to the following questions were retained:

(1) how do humans and goats communicate?
(2) which are the factors affecting human–goat relationship?
(3) how can we measure the quality of human–goat relationship?

Papers that did not answer to these questions (e.g., papers dealing with intra-specific communication or interspecific interactions not focusing on goats and humans) were considered non-relevant for the present review, and were therefore eliminated.

**Figure 1:**
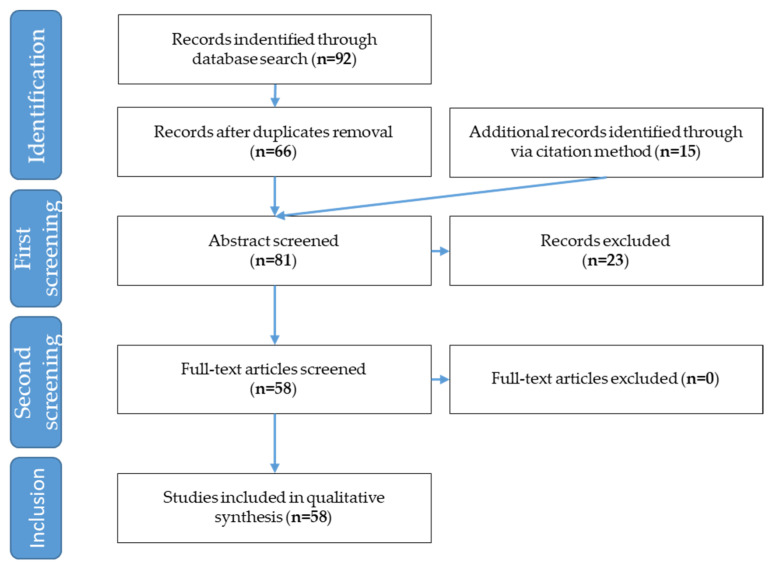
Flow chart of the systematic literature review process displaying exclusion and inclusion steps.

After this screening, 58 articles were retained for full-text reading (Figure 1) and imported in the reference manager and text editor Citavi^®^, where the structure of the chapters was created. All these articles focused on domestic goats, except for one that was on feral goats. No articles were retrieved on the interaction between humans and wild goats.

## 3. How Can Humans and Goats Communicate?

A fundamental assumption in order to establish a positive human–goat relationship lies in the human ability to understand signals emitted by goats and in the awareness of how goats react in response to human behaviour [47]. Therefore, communication between humans and animals influences the development of the HAR [2]. Furthermore, referential and intentional communication can provide cues which stimulate socio-cognitive processes [47].

It has been suggested that, during the domestication process, animals developed different levels of specialisation in the field of inter-specific communication with humans [48]. Compared to other species which have been domesticated mainly as companion animals, such as dogs and horses, goats, as with other farm animals, may be expected to have lower skills for communicating with humans [47], because selection during the domestication process has pointed mainly in the direction of decreasing emotional reactivity towards humans and increasing production traits, such as milk yield or weight gain, but not towards the development of a direct cooperation with humans [48]. Nonetheless, based on a study on the ability of goats to identify human cues, Kaminski et al. [49] hypothesized that the capacity of goats to react to human cues, even in the absence of specific training, might be a side-effect of the ancient domestication of this species. Recent research suggests that human–goat communication can also be seen in the frame of a broader posthumanist linguistic framework, and therefore, that interspecies interactions may be included within the traditional linguistic theory [50]. In fact, although goats do not use verbal communication, humans can speak to them and can learn to understand their minds, consequently adapting their behaviour towards the animals [45].

Communication, either intra- or inter-specific, plays a fundamental role in social relationships and has many functions, such as localizing and identifying other individuals, gathering information on food or shelter location, sending commands, establishing or strengthening the social status, establishing or maintaining social relationships or signalling a temporary physiological state (e.g., oestrus signals). Communication signals can be either visual (e.g., postures), acoustic (e.g., vocalizations), olfactory (e.g., emission of particular odorous substances, such as pheromones), gustative or tactile [51]. For the scope of the present review, only intentional communication signals between goats and humans (and vice versa) will be reviewed, and effective intentional communication signals retrieved from 23 relevant papers are summarized in Table 1.

### 3.1. Olfactory Communication

Olfactory signals are particularly important for intra-specific communication in goats [66] and a wide range of odour signals is produced to send, for example, sexual (e.g., [67]) or maternal cues [68]. However, there is only very little evidence about the use of olfactory signals in human–goat communication. Only one of the articles included in the present review reports smelling behaviour, probably with the aim of obtaining olfactory cues, directed from goats towards a human being, which in this specific case was a person who periodically massaged the animals [56]. The reason why so few studies report information on olfactory communication between goats and humans is probably related to the common belief that humans’ olfactory abilities are not particularly developed compared to those of other mammalian species. However, recent research aiming to compare human and animal olfactory capabilities demonstrate that this belief is not grounded on sound scientific data. In fact, in [69] suggests that humans have a well-developed sense of smell, which regulates a variety of human behaviours and that, in many cases, is more developed than that of several animal species. Therefore, the field of human–goat olfactory communication probably deserves further attention to gain scientific evidence about this communication means.

### 3.2. Visual Communication

Visual communication can be used as a means for transmitting specific cues, and has therefore been often investigated in cognitive studies. Goats are able to correctly interpret visual cues from humans, such as pointing the arm towards a given direction, touching a given object or slowly moving arms and hands [49,55,63], even though the effectiveness of pointing the arm seems to be affected by the distance between the arm and the reward [64]. Body orientation is also an effective visual communication signal [36], whereas hand position (in front of or back the experimenter) and head orientation alone do not convey relevant information to goats [36,63]. However, human’s head in combination with body oriented towards the goats seem to stimulate the attention of dwarf goats, that show active anticipatory behaviour for a reward (i.e., food), regardless of the fact that the experimenter’s eyes are open or closed [62]. On the contrary, later studies [36] showed that open eyes may provide subtle cues to goats, which seem to prefer approaching experimenters with open rather than closed eyes. Other interesting effective cues are facial expressions: ref. [7] demonstrated that goats seem to prefer interacting with happy rather than with angry human faces. 

Contrasting results were found on the use of human gaze to communicate with goats: in fact, in [61] found that gazing was effective to indicate a direction to goats, whereas [49] did not report successful results on the use of human gaze to provide cues for locating a hidden reward.

A postural signal, often used also on farm to attract goats’ attention, is the action of offering food, as reported by [50] in a petting farm or by [46] in a pet-therapy experiment. This signal was also used by [65] in a cognitive test, where the experimenter shacked a container with dry pasta to motivate the goat to walk towards the food. In this study, the noise produced by the food shacked into the container probably also acted as acoustic stimulus to attract the attention of the goat. 

From the goats’ side, in [60] observed that goats gaze at humans when they are searching for cues in order to get a reward, but they also noted that this behaviour was more pronounced when the experimenter was facing towards them rather than when he was oriented backwards. Gazing at humans was also observed in unsolvable task test studies, where goats alternatively looked at the experimenter and at the inaccessible food reward, as if they were looking for cues to solve the task [48,57]. In contrast, in [58] could not confirm an increase of gazing duration nor a decrease of latency to gaze in goats searching for help to reach a food reward.

Other reported visual signals include body postures, such as goat kids turning their head and directing their gaze away from the milk bottle that humans are using to feed them, in order to show their lack of interest to be milk-fed [50]. Similarly, in [53] describes a goat turning its neck/head and moving it 90° to the left, right, up or down, or moving away from the trainer, in order to show its refusal to wear a halter. On the contrary, in the same study, the goat stood in front of the trainer or moved toward him to establish a positive contact. Approaching a standing human was also performed by goats during a test in order to establish contact [55].

In conclusion, these findings demonstrate that goats can successfully interpret some physical cues and visual signals from humans, such as body posture and, to a less extent, head orientation, and they seem to be sensitive also to subtle cues such as open eyes and facial expressions. On the other side, goats send visual signals to humans to seek for help. Furthermore, it is worth noting that visual signals may be particularly effective in the communication between goats and children because, being goats a medium-sized species, their face is close to that of children, who can therefore better observe goats’ facial expressions and easily interact with them [45].

### 3.3. Acoustic Communication

Goat are a highly social and vocal species, exhibit vocal plasticity in response to the social environment [70], can individually recognize conspecifics’ vocalizations [71] and are able to distinguish the emotional valence of conspecifics’ vocalizations [72]. Therefore, vocalizations can be good indicators of the sender’s characteristics, such as identity, body size, age, hormonal status and affective states, and a large amount of studies have been carried out on intra-specific communication in goats (e.g., [26,70,72,73,74,75]).

Nonetheless, to our knowledge, no studies have specifically taken into account aspects related to inter-specific vocal communication between humans and goats or between goats and humans. However, some vocal signals directed from humans to goats have been described, such as the use of vocal calls like “Come here” or “Come on, honey” emitted by humans in order to attract a goat’s attention [36,50], or anecdotic sentences like “Hmm, that’s tasty huh?”, emitted to invite goats to receive food on a pet farm [50]). These examples support the hypothesis that humans tend to be rather anthropocentric in their relationship with goats, addressing the animals using human language [50]. The human voice has also been used in association with different handling treatments [23,54,55], although specific studies aiming to identify the role of the characteristics of the human’s voice on goats’ reactions are lacking and certainly deserve attention for future research.

A particular acoustic stimulus is represented by a bridge stimulus, such as the sound of a clicker, used during operant learning procedures in training programmes. For example, a clicker sound that had previously been associated with a food reward was successfully employed by [53] to train goats to wear a halter and to be led by the halter.

Similarly, the use of acoustic signals at different tones was used by [52] in association to a positive reinforcement (i.e., water): lower tones (440 Hz; 80 dB) were associated with the reward, whereas higher tones (980, 1039, 1166 Hz; 80 dB) were not. This acoustical secondary reinforcement was effective to convey information to the goats about the right shapes to choose in a test to get the reward.

Apart from the above-mentioned reports, no other inter-specific vocal signal was retrieved from the selected papers.

Nonetheless, from the existing literature on intra-specific vocal communication in goats, we may find useful cues to assist humans in interpreting goats’ emotions by listening to their vocalizations, even though these vocalizations are not intentionally human-directed. For example, vocalizations related to positive emotions, such as the expectation of a food reward, show a lower frequency modulation extent and a less pronounced fluctuation in fundamental frequency of both closed- and open-mouth bleats, whereas in situations of high arousal, calls present higher fundamental frequencies and higher energy distribution, with a more pronounced variation [76]. For example, alarm vocalizations consist of high-pitched sneezes [77]. Vocalization characteristics of farm species, including goats, have been recently reviewed by [78], showing that some kinds of vocalizations are related to specific contexts: in goats, for example, guttural noises (gobbles) and snorts are related to sexual behaviour and bleats play a role in maintaining social relationships inside the group, whereas open-mouth bleats are usually related to negative situations, such as pain, or in some cases, may also be emitted in positive contexts, such as when goats are waiting for a food reward.

Anecdotic information on the ability of humans, including children, to perceive differences in goats’ vocalizations is provided by [45], who report that, after the replacement of a goat, the teacher and children of a primary school noted these differences in their goats kept for educational purposes, and “wondered what this meant” [45].

Given that goats are highly vocal animals, further studies on the ability of humans to recognize the meaning, valence and arousal of goats’ vocalizations would greatly help to strengthen and improve human–animal relationship. In the future, it would also be interesting to develop methods, such as smartphone apps for simultaneous decoding of multiple vocal parameters, to automatically identify the meaning of goats’ vocalizations [78].

### 3.4. Tactile Communication

Tactile interactions are frequently used by goats for intra-specific communication (e.g., grooming, fighting [66]), but they can also be observed in inter-specific communication between goats and humans, for example, when goats are facing an impossible task to solve, especially if they have previously established a positive HAR [48,57,58]. In these studies, goats have been observed to look for physical contact with humans, such as rubbing, nosing, pawing a hand or leg or jumping up, probably in order to ask for cues or help to solve the problem and reach the expected reward, as described above for visual signals. Mastellone et al. [57] considered this tactile approach as a socio-positive (or at least neutral) behaviour, since no antagonistic events were recorded in his study. 

A positive tactile communication was also observed by [56] in goats submitted to a massage treatment, where the animals were observed to look for contact with the masseur by rubbing their head and placing it on human’s lap, and also nibbling the masseur, although this last behaviour probably also involved a kind of gustative communication. Goats also exhibited some contact behaviours that were considered as having a negative valence, such as biting and pulling human’s clothes or pushing the human’s arm and hands with head or horns, which might be associated with a feeling of frustration possibly generated by an association between the masseur and the feeding period that induced the onset of aggressive behaviour [56]; similarly, chewing behaviour, consisting of the contact of the goat’s mouth with a person or an object (other than food) on a person, was also considered as a negative reaction to a human being (trainer) trying to convince the goat to wear a halter [53]. It is possible that this behaviour may convey some information by means of gustative communication, besides tactile communication, although from this study we cannot draw any conclusion in this respect.

From the human’s side, in [56] the masseur established a tactile communication with goats by means of a massage that was aimed at promoting goats’ relaxation and improving the quality of HAR. Similarly, in [59] proposes that brushing the goats’ head and back may have a positive effect on the animals’ emotional state. Petting, stroking and scratching the goats were also effective for achieving a positive handling treatment [19,23,54]. Tactile signals (touching, stroking, petting, scratching and brushing) were also used by disabled persons to establish a contact with goats used for a rehabilitation therapy [46], and by familiar and unfamiliar humans in a handling test [55], demonstrating that goats are sensitive to these kind of signals.

### 3.5. Gustative Communication

Very little information is currently available on the use of gustative signals in human–goat communication. Some evidence is reported in one article [56] where goats are described as licking and nibbling a human person, and this was interpreted by the authors as a positive search for contact with humans. 

Anecdotal reports of nibbling of non-food items, including, for example, humans’ clothes, have also been reported by [50], although their meaning and effectiveness are not clear: the authors interpreted them either as a mean to look for social interactions, or as a replacement behaviour performed by animals living in a poorly enriched environment.

## 4. Factors Affecting Human–Goat Relationship

The factors that can influence the quality of HAR can be traced back to both animals and humans. As far as animals are concerned, both environmental and individual factors may affect HAR. Examples of environmental factors are the type of farming system in which animals are raised [54,79], or the handling experiences that generate expectations on the interaction with humans [1]. As for individual factors, the animals’ genetic makeup explains the differences in temperament and therefore in docility towards humans [80], while other individual goat characteristics, such as social rank, explain behavioural differences towards humans [81]. Furthermore, humans’ characteristics, such as attitudes and empathy toward animals, should be taken into careful consideration, as they determine the behaviour of the stockpersons when working with the animals, and can therefore influence the quality of HAR [82]. Empathy, in particular, underpins attitudes towards animals and has a profound influence on animal fear towards humans, which in turn may affect their performance and welfare [33,82]. Other human characteristics that play an important role in this sense are represented by the propensity to work with animals, the knowledge and technical skills of farmers [82] and their commitment and job satisfaction [33]. Beaujouan et al. [83] also underlines that it is mandatory to consider the work context in which stockpeople operate, in order to fully understand how the HAR develops. The human factors which influence the human–goat relationship are described in the “human perspective” section of this chapter. All the factors identified through the 17 retrieved articles are summarised in Table 2.

### 4.1. Environmental Factors

The social and physical environment are important factors, which act on the nature of HAR. The social environment is particularly important, as learning to distinguish acceptable from unacceptable social behaviours is part of the normal socialisation process and development of social skills [1], which occur mainly in young animals, especially during the sensitive period for the establishment of this relationship [54]. For these reasons, early handling experiences and early contact with humans have a particularly marked effect on HAR and, according to a number of studies, positive interactions between humans and goats should be established as soon as possible within the farm and the results are particularly effective if performed with newborn kids. In fact, as goats are a precocial species, kids are able to interact with humans since the beginning of their developmental stages, thus allowing an early establishment of social bonds [57]. It has to be underlined that the effect of early socialisation may persist throughout time: for example, in goats, the persistence of the effect of human contacts is supposed to range between 8 [54] and 25 months [17]. 

Five studies investigated the role of human rearing of goat kids, including early contact and manipulation, on HAR. The first study dates back to 1988 [80]. Lyons et al. [80] compared the behaviour of goat kid twins, in which one was raised by the dam and the other by humans, finding that different early experiences led to different behavioural expressions of temperament that tended to remain stable and persist over time until 30 weeks of age. In particular, human-reared kids exhibited a significantly higher amount of time in proximity with humans, a lower latency to approach them and a lower flight distance compared to dam-reared kids. [17] also noted that different previous experiences in terms of frequency of opportunities for human contacts led to different goats’ reactiveness in encountering an unknown human being as a novel stimulus, in a home pen. In particular, multiparous goats raised by their mothers showed a greater human avoidance behaviour, as suggested by the longer latency to approach and lower time in proximity with humans compared to multiparous goats separated from their mother at birth and bucket-fed 2–3 times/day for 10 weeks by humans. This was also accompanied by a reduction of milk ejection in dam-reared goats. Similarly, a study by [84] showed that, five weeks after weaning, artificially-reared kids showed shorter avoidance distances from humans than dam-reared kids. Toinon et al. [84] therefore suggests that bucket feeding, accompanied by positive physical interactions (e.g., gentle stroking), allowed one to establish a good HAR. Finan [85] also noted that, in an artisanal farm in the United States, the isolation of goat kids from their mother and bottle feeding allowed developing a very strong relationship between caretakers and baby goats. This experience of caring for goat kids represented an opportunity for caretakers to interact with them by calling them by name, a name the kids learned. By learning to trust humans and to enjoy their presence, the kids became more friendly toward humans and, therefore, easier to manage in adulthood.

However, artificial feeding (i.e., feeding with a multi-nipple bucket) in itself is not enough to shorten kids’ flight distance, even when milk is delivered by caretakers. In fact, Boivin and Braastad [54] found that these factors (artificial feeding provided by the caretakers) were effective for improving HAR only if goat kids had also previously been isolated from peers for a short period (i.e., 10 min/day for 10 days) and handled by humans. The authors argued that a short period of isolation and handling at an early age is sufficient to reduce kids’ flight distance, at least up to 7.5 months of age. 

In addition to the early interactions between humans and goats, the number and quality of these interactions is also important for the establishment of the human–animal relationship. This has been addressed in 5 articles. Mastellone et al. [57] highlighted how frequent interactions with humans in a zoo led to a higher number of human-direct behaviours in goats. In this study, the authors found that, in an impossible task paradigm, goats with a long history of socialization (i.e., presence of visitors in the home pen almost daily) performed a higher number of visual, tactile and approach behaviours directed towards humans, compared to goats accustomed to the simple routine care by the zookeeper. In particular, tactile behaviours (i.e., rubbing, nosing, pawing a hand or leg or jumping up) were significantly more frequent in the socialised group. Furthermore, Miller et al. [22] demonstrated that more frequent interactions with humans, represented by entering the goat enclosure daily and walking calmly, were effective in favouring the adaptation of feral rangeland goats to intensive farming systems. In fact, these frequent interactions led to less aggressive reactions and reduced flight responses compared to those recorded in feral rangeland goats under similar farming conditions, which had experienced a lower degree of interactions with humans. In line with these findings, Yoshida and Koda [58] found a shorter latency to the first contact in intensive farms compared to semi-extensive farms, probably because intensively reared goats are more accustomed to contacts with different people.

A gentling treatment (stroking goats’ back and neck with eye contact for 10 min for 24 days) was applied by [19] on adult dairy goats, resulting in animals that approached the experimenter more quickly in a latency test, and which habituated faster to the presence of the experimenter. However, Langbein et al. [48] suggests that a gentling treatment (friendly talking, gentle touching, stroking and hand feeding) conducted for a limited period of time (twice daily, over two weeks) may not lead goats to interact more with humans. Interestingly, the authors highlight how standard husbandry care in a farm context may be sufficient to ensure that the goats show referential and intentional behaviours directed towards the human being (alternation of gaze and contact) in difficult contexts (i.e., unsolvable task paradigm). 

The physical environment in which animals live may also have an impact on the quality of HAR. For example, a study conducted on 30 Italian farms showed that the farm size significantly affected the percentage of goats that accepted being gently stroked on the head for >3 s by an unfamiliar person during the AD test. This percentage was higher in small farms (<50 lactating goats) than in large farms (>100 lactating goats); the percentage of goats that accepted to enter in contact with the unfamiliar person, but withdrew within 3 s, was also higher in small farms, although differences were not statistically significant [88]. The apparently better relationship of the stockpersons with goats in small farms might be due to lower workload in these farms, with a more favourable ratio between the number of goats and both the number of permanent workers and the number of milkers. It is therefore possible that having more time to dedicate to each goat may favour the creation of a more positive HAR. However, these results were not confirmed by [91], who did not find significant differences in the latency to first contact test between small and large farms in Portugal, although the latency was unexpectedly lower in larger farms (but this was attributed to goats’ individual traits, as explained in the next paragraph). 

Miranda-de la Lama et al. [81] showed that the presence of environmental enrichments, represented by a raised feeding, physical barriers and elevated areas (i.e., two tractor tyres, a heap of compacted earth), significantly affected the behavioural and physiological responses of dairy goats to manipulation and restraint. In fact, goats in the enriched environment showed higher blood cortisol levels and remained further away from the experimenter, compared to control goats, probably due to the greater possibilities of modulating the distance from humans in this environment. In the enriched environment, episodes of aggression against the experimenter were also more frequently observed, the causes of which remain to be investigated. A higher frequency of defence behaviours was recorded in control goats, which opposed greater resistance to capture, probably because of the lack of elements for hiding in the pen.

### 4.2. Individual Traits

In addition to environmental factors, genetic traits may also affect the HAR, for example, due to individual differences in terms of temperament, which are relatively consistent over time. Temperament determines goats’ attitude towards humans, and goats’ behavioural reactions. For example, a significant correlation was found between goats’ individual temperament (expressed as “timidity” score, where goats that were more tense, watchful, excitable and fearful of people, and less friendly toward people, had the higher scores) and the response of goats to a set of five behavioural tests, including latency to proximity and latency to contact to several test objects, including a human being. These behavioural differences seem to be related to different pituitary adrenal responses [17,80].

Genetic differences also exist, for example, among goat breeds. Molale et al. [89] observed that Tswana goats responded more negatively than Boer goats to human manipulation. In particular, differences in flight time and speed between the two breeds were attributed to differences in goats’ temperament, with higher flight time (i.e., the time required for a goat to travel a distance of 4 m after being released from a crush pen) and flight speed in Tswana goats, which were then considered more fearful of humans than Boer goats. According to the authors, the calm temperament of Boer goats would make this breed easier to handle. In [90] also found a calmer temperament in Boer goats, as well as in their crossbreds, compared to more “combative” breeds, such as the Nguni and Xhosa lob-eared in a context of manipulative practices (blood sampling and rectal palpation). Notably, Nguni and Xhosa goats exhibited a higher flight speed, a higher vocalization score and a more pugnacious behaviour when put into a crush than Boer goats and crossbreds. Moreover, Nguni and Xhosa goats’ flight time resulted shorter than those of the other breeds. Temperament differences are suggested to affect the HAR also in the study by [91], who hypothesise that the better HAR that they recorded in large farms may be due to the higher prevalence in these farms of breeds like Saanen and Murciano-Granadina, which are more docile and easier to handle compared to the more rustic breeds bred in smaller farms, which are known to be more suspicious.

Social rank may also affect goats’ relationship with humans. Miranda-de la Lama et al. [81] observed that higher-ranking goats maintain a larger distance (half a meter more) from an experimenter positioned in the centre of a box for two minutes, compared to lower-ranking goats. The authors explain this behaviour based on the considerations of their previous study, according to which goats with different dominance profiles would face the human presence differently and use different social strategies [94]. They also suggest that low-ranking goats get used to the stress induced by handling and restraint earlier than high-ranking goats, due to a passive profile in these low-ranking animals [94]. Despite this, goats with a low-ranking position required more time to be captured, and showed a higher number of aggression episodes toward humans, the causes of which still have to be investigated [81].

### 4.3. Human Perspective

Human–domestic animal relationships are inevitably unequal, involving human management and control of animals, either companion or farm animals. 

A growing number of studies carried out on farm species such as pigs, cattle, horses and even alpaca and llama show that the farmer’s or stockperson’s characteristics play an important role in the welfare of these animals: in particular, personality, attitudes, empathy levels, beliefs in animal mental capacities are reported to affect behaviour towards the animals, concern for animal welfare and decisions on housing and management [95,96,97,98,99,100,101]. In addition, a number of sociodemographic variables, such as gender, age and education level and previous experiences with animals affect the way in which animals are considered, treated and cared for (e.g., [102,103,104,105]). Hemsworth and Coleman [98] pointed out the importance of positive attitudes and empathy of stockpersons toward animals, even in intensive production systems, and their role in reducing fear and stress. In fact, the way in which farmers think about their animals and feel toward them appear to be associated with the way they behave towards them.

One central characteristic for building a positive human–animal relationship is empathy, which can be considered as the ability to understand, share and care about another individual emotional state [106,107]. Empathy toward animals is associated with empathy toward people [108], and with positive attitudes toward animals and their welfare [105,109]. For example, Kielland et al. [110] found that dairy cattle with fewer skin lesions belonged to farmers with higher levels of empathy toward animal pain. Similarly, Norring et al. [111] reported that empathic veterinarians scored cattle pain higher. 

In addition, attitudes towards animals, broadly defined as psychological tendencies to evaluate a particular entity (e.g., other humans or animals) with some degree of favour or disfavour [112], contribute to determining animals’ health, welfare, productivity and management by affecting farmers’ behaviour. 

Among negative emotions, the perception of pain is certainly of primary importance, and [113] observed that stockpeople who believe goats are pleasant animals are more likely to worry about the pain they feel. The study revealed that females, older farmers and those raised in a rural district turned to the veterinarian more frequently. The perception of goats’ pain by humans was also negatively associated with living on a goat farm, having agriculture as the main income and having experience in recognizing cases of pain. However, the authors highlight how the therapeutic decisions made by individuals can be different depending on the individual’s tendency to engage in helping behaviours. In another study, Muri et al. [92] explored empathy and attitudes towards goats in the Norwegian dairy goat industry and the influence of some demographic variables. The authors tested 260 Norwegian goat farmers using multi-item rating scales specifically developed to assess attitudes and empathy toward goats. They found that the three empathy factors that emerged in their study (i.e., Emotional contagion, Personal distress and Perspective taking) were significant predictors of attitudes towards goats: each empathy dimension was associated with a different attitude factor. In particular, farmers who obtained higher scores in the empathy dimension labelled as “Emotional contagion” (i.e., emotional response to interaction with goats) showed a more positive attitude towards how pleasurable it was working with goats. Farmers scoring higher on the “Perspective taking” scale (i.e., taking the perspective of others regarding their emotions about goats) had more positive attitudes towards the general characteristics of goats; finally, farmers scoring higher on the “Personal distress” scale (i.e., self-oriented distress when observing goats in negative circumstances) were more positive towards the ease of working with goats. 

Different dimensions of attitudes and empathy were associated with different demographic variables including gender, age, having grown up with a companion animal or a horse or in a rural district. Gender differences were observed in both the “Perspective taking” and “Personal distress” dimensions, with women scoring higher than men. Women also showed more positive attitudes in relation to the general characteristics of goats and the ease of working with them. Having a companion animal or a horse in childhood was positively associated with “Perspective taking”, while education was negatively associated with “Perspective taking” and “Emotional contagion”. Farmers having grown up on a farm were less in agreement that goats are pleasant animals to work with, fun and intelligent. Farmers’ attitude towards goats, and in particular, their belief in the importance of positive contact with them (e.g., stroking), was found to be linked to the behaviour of the goats in a test in which the animals were restrained and an unknown person tried to touch their chin [93]. 

In evaluating the validity and feasibility of various tests for HAR evaluation in goats, Mersmann et al. [87] studied the attitudes and behaviours of farmers towards goats. These were found to be associated with the different avoidance and approach tests used, where a higher proportion of negative interactions during milking (i.e., harshly talking, hit and kick the goats during milking) were associated with higher avoidance and lower approach behaviours in goats.

## 5. How Can We Assess the Quality of Human–Goat Relationship?

Besides the number of reviews that investigated the importance of positive interactions between humans (e.g., farmer, stockperson, milker) and farm animals (e.g., [1,2]), it is argued that measuring the positive relationship is difficult in animals and that it is more difficult to measure positive rather than negative relationships [1]. To this aim, Rault et al. [1] listed potential indicators that can be used to evaluate the quality of the relationship between humans and farm animals (e.g., behavioural changes, physiological changes). 

Tests and indicators usually measure the reaction that animals have in response to a human’s presence. However, a number of confounding effects can affect the results; hence, caution should be made when inferring a positive or negative relationship, as the response of animals can be influenced by several factors. According to [1], the voluntary approach of animals towards humans is a sign of positive relationship or curiosity, but the absence of seeking this interaction is not necessarily a sign of a negative relationship. The interest towards humans may sometimes depend on the context (e.g., indoors or outdoors). The motivation to the stimuli can also determine the reaction of animals towards the human. For example, when performing tests during the feeding time, the interest that animals show towards humans is reported to be low in dairy cattle because of other activities occurring at the same time (e.g., feeding, but also competition). A further confounding effect is the evaluation of social animals performing tests in a test arena with isolated animals: the results of these tests should be carefully evaluated since stress due to handling, isolation or novel environment can conceal the reaction to humans due to stress [2]. The interactions with humans are also species-specific and, within each species, breeds and attitudes can also act as confounding factors, determining the choice of the most appropriate test: e.g., local breeds are more responsive to humans and animals reared in extensive systems may avoid humans more than those reared in intensive systems since they are less accustomed to the constant and close presence of the farmer [91]. This aspects stress the importance to develop measures specifically validated for a species or breed in a specific context and discourages from using measures tested for other species or under different circumstances without prior validating them for the specific context of intervention [114]. Therefore, one of the aims of this review was to present an overview of potential measures of HAR quality that have been specifically applied to goats in different contexts.

### 5.1. Behavioural Tests

Behavioural tests measure the reactions that goats show towards human beings. After the first reaction, namely, orientation response [1], goats can react to humans showing a withdrawal behaviour and avoiding contacts, approaching the assessor and seeking for interactions, or standing still. Most of the tests can be classified according to three main categories: (1) reactions to a stationary human, (2) reactions to a moving human and (3) responses to handling. The assessor that conducts the test can be either familiar (e.g., farmer, milker) or unfamiliar (e.g., researcher, veterinarian) to goats. The test setting can be the home pen or a test arena; tests can be performed isolating the goats from the familiar group or together with peers [2].

Table 3 summarizes the behavioural tests developed so far to evaluate the quality of human–goat relationship.

Eighteen papers were extracted during the literature review, with a total of 28 tests identified. Results equally split in tests that involve assessors moving in the pen, usually approaching individual animals, and stationary tests, with the assessor motionless in the environment with goats (both 39.28%). Only one stationary test requires that the assessor moves the hand to touch the animals [93]. Only two tests can be classified as handling procedures [17,93]. Both these last tests do not really imply an experimental handling, but the judgement or self-evaluation of the behaviour of goats during milking; hence, the handling refers to the way the stockperson behaves, touches and interacts with the animals. The remaining tests are classified as both stationary and moving human as they are normally composed of sequential phases [17,116]. The evaluation of the quality of the human–goat relationship has commonly been performed by unfamiliar assessors (67.86% of the tests) and preferably in the home environment (67.86%). The last attribute of the tests is the social context: 71.42% of tests have been performed with goats in their familiar group, even if some measures can be collected on individual animals. Only in one experiment, goats were individually tested but with peers in an adjacent pen with an open wire fence [115].

The absolute number of tests identified during the literature review does not correspond to unique tests. Indeed, many papers describe the same test with only little rewording of the description of the test or few changes. For example, [88,91,118] included a simplified version (e.g., less variables collected) of the latency to the first contact test validated by [117]. In turn, the same authors [117] applied a shorter version of the test developed by [19] (the test proposed by [117] is capped after 5 min if no contacts with goats occur, whereas the original test [19] is capped after 10 min). Before these recent studies, to our knowledge, behavioural tests measuring the latency to the first contact both in adult goats and kids were originally developed by [17,80,116]. Mersemann et al. [87] also collected the latency to the first contact, but the period the assessor remains motionless in the pen was increased to 15 min. Where tested for validity (Table 3), latency to the first contact test resulted valid, except for [87]. The duration of the test, whether capped after 5 or 10 min, depends mainly on the need to have feasible indicators, but it does not alter the validity of the test. When [117] validated this test, a large difference between farms labelled as “good HAR” vs. farms labelled as “poor HAR” was found (good: 8.0 ± 4.7 s; poor: 136.0 ± 55.2 s). The latency to the first contact test is generally reported as feasible: no specific training is required and time devoted to this procedure is limited. Furthermore, this test rarely induces fear or stress in animals [117]. The only suggestion made by the authors is to perform this test without the presence of bucks when evaluating the quality of HAR in dairy goats [117], because, in different trials, bucks were found to be the first to approach the assessor with females standing still in the pen. After removing the male, the authors found that the behaviour of females changed and approached the assessor. Mersmann et al. [87] also found that hornless goats show shorter latency compared to horned goats, probably because they respect the social distances among goats less than the latter and more easily make their way through the group to reach the assessor. After having been tested on farm, the latency to first contact test was included in the AWIN welfare assessment protocol for goats [41,88,91,122]. Based on the results of these preliminary on-farm tests, the AWIN protocol also contains indications that, if more than 24 s elapse before any contact occurs between goat and assessor, the HAR should be considered sub-optimal [41].

Tests belonging to the moving human category are largely represented by avoidance distance tests performed in the pen on individual animals in the group with peers. However, again, individual animals were approached using with similar procedures [41,91,114,117]. Differences are mainly related to the starting distance in front of individual goats to begin the test: this is 300 cm in [114], similarly to tests performed in dairy cows, whereas it is reduced to 200 cm in [41,91,117]. A larger starting distance (20 m) is proposed by [22], but this decision is justified by the objective of the assessment: the research investigated the habituation of feral rangeland goats to intensive farming systems, whereas [41,91,114,117] studied the reaction of dairy goats in commercial indoor farms. Even if the validity of the avoidance distance test was confirmed by [117], its application in large groups is likely not feasible, and many authors do not support its use, especially in on-farm situations (e.g., time consuming, extensive training required [117]). According to [117], the avoidance distance test is less suitable for goats than for cows, because of the different management. Dairy cows are accustomed to humans frequently entering the pen for cleaning operations, whereas goats are unlikely to experience close contacts, since the housing on deep bedding mainly involves the farmer topping up the pen with straw, frequently from outside, and rarely entering. During the testing of the prototype of the AWIN welfare assessment protocol for goats in Portugal, Can et al. [91] found that the feasibility and also the result of the avoidance distance test can be influenced by breed (e.g., local breeds appear to be more reactive) and production system (e.g., intensive vs. semi-intensive)). The results obtained by the authors do not allow understanding if the test is not suitable for certain breeds or husbandry systems (or an interaction between these two aspects) or if the differences found are the result of a lower familiarity with humans in local breeds. Contrary to the experience reported by [114], Muri et al. [93] could not apply the avoidance distance test in the pen, as goats flocked around the assessor, making the evaluation impossible. Mersmann et al. [87] reinforced this assumption and added that this method is hard to be used if many shy animals are present, because they behave as a whole group, making it unfeasible to perform an individual assessment. Furthermore, the individual identification to avoid retesting the same animal is difficult, since goats usually have small ear tags with small identification codes or numbers. Can et al. [123] also found low consistency over time (from winter to summer, approximately four months between the two assessments). Mersmann et al. [87] tested and validated an avoidance distance test in the pen with dairy goats with two successive step. In the first step, the assessor only records the average distance of the group, considering the closest goat. In the second test, the assessor approaches individual goats and tries to touch them. The authors report that this test is feasible, not affected by external factors, and clearly related to the quality of the previous interactions between humans and goats (e.g., frequencies of contacts).

Moving human tests can also be performed at the feeding rack. Mersmann et al. [87] tested two procedures: the first involves the assessor walking along the feeding alley, recording the number of animals that continue feeding when the assessor passes by. The second resembles the avoidance distance test at the feeding rack already used in cattle, with the assessor approaching individual goats during feeding and measuring the individual avoidance distance. The authors reported that the feasibility of both avoidance tests at the feeding rack is supposed to be high (e.g., only 9 min for the assessor to pass by the feeding alley and record the number of feeding animals). However, due to some constraints (e.g., design of the feeding alley, presence of feeding belts), those tests were only applicable in the 70% of farms [87]. Some refinements could be made to support the use of the test involving the assessor walking on the feeding alley, as this test well describes the quality of the relationship with humans and, contrary to the findings in dairy cows [124], is not affected by confounding factors.

Muri et al. [93] applied a test that is classified as stationary human since the assessor does not walk towards goats, but stands still in front of individual goats and tries to touch the chin of the animal. However, reaching a goat to begin the test implies a sort of approach that can elicit a fear reaction, increasing the flight distance. Indeed, Muri et al. [93] reports that this test was only performed in half of the 30 farms evaluated.

Most of the tests were validated indoors, sometimes in a test environment, but also in the home pen. External validity was also confirmed in some cases where the application of test was made in commercial farms [87]. However, information on the use of behavioural tests to measure the quality of HAR in outdoors and in extensive husbandry systems is scarce. It is known that a reduced number of contacts during the whole year or in a limited period (e.g., summer grazing) can affect the perception and familiarity that animals have with humans (e.g., dairy cows after summer pastures show greater avoidance distance than before the grazing period [125]). The Familiar Human Approach Test (FHAT) included in the AWIN welfare assessment protocol for sheep [126] was used by [119,120] to evaluate the human–goat relationship in extensive systems. This test requires that the farmer behaves in the normal way, gathering the animals for inspection, and an assessor evaluates the behaviour of goats and the distance from the assessor. This test has never been validated in sheep [127] and has not yet been validated for goats either. However, Battini et al. [119] report that the execution of the test is feasible and reliable among assessors; hence, it is a promising indicator to be included in a welfare assessment protocol for goats in extensive farming systems, after its validation. Muri et al. [93] applied a similar test indoors: the farmer was asked to select 20 goats and to mark them as a part of the usual routine. Then, the assessor evaluated both human and goat behaviour on a five-point scale. This test provided interesting results; however, its repetition may yield biased results, because the farmer’s behaviour may be affected by knowing that his/her behaviour is being observed and scored during the test [93].

### 5.2. Other Behavioural and Physiological Indicators

In addition to behavioural tests, other indicators have been used to measure the quality of HAR. Most of the indicators presented in this section are not feasible in on-farm situations but can be included in experimental settings. 

Information that vocalisations provide about animals’ emotions has been gathering increasing attention and has been addressed in recent studies in different species, including goats [78]. Goats may bleat with open and closed mouth. According to the limited research conducted so far, it is known that open-mouth bleats can be produced during both positive (e.g., anticipation of food) and negative (e.g., pain) situations, and are used to maintain social contacts with peers. A reduction in distress vocalizations, supported by increased proximity to humans, was interpreted by [54,115] as a positive perception of human presence. However, none of these studies provides information on the underlying emotions, but only changes in rate of occurrence of vocalisations. Goats also produce alert sounds. Alarm snorts, also called sneezes by [117], are described by [66] as a loud, high pitched, short and abrupt closed-mouth sound that goats produce to warn the group of a possible danger. Farmers are aware of the meaning of this sound and fostered research on this topic. Therefore, Battini et al. [117] tried to test the validity of this indicator, but unfortunately, the frequency of occurrence of sneezes in their study was too low to allow drawing any conclusion in this respect.

Among physiological parameters, oxytocin can be related to the perception that animals have of humans. Positive interactions with familiar humans are known to raise oxytocin concentration [1]. Oxytocin concentrations can be measured from saliva samples, which present the advantage that they can be easily collected in a non-invasive way; however, cost constraints for further analysis reduce the feasibility of this method in the on-farm situation, and make its use more suitable for research purposes [128]. Nevertheless, Lürzel et al. [55] could not confirm the validity of this indicator in goats, because they found a negative association between oxytocin concentration and animals being stroked by a human. The result is surprising, since goats freely chose to approach the human, but probably they did not perceive the interaction as positive. Some authors suggest that the situation, more than the action itself, may trigger the release of oxytocin [129]. Lürzel et al. [55] speculate that their unexpected results may be determined by the fact that goats were separated from their kids to perform the test, and were therefore experiencing a negative emotional condition, or by the physiological state of the animals (e.g., lactation). The low sample size of this experiment (n = 9) might also be responsible for these unexpected results. 

### 5.3. Attitudinal Questionnaires

Attitudinal questionnaires developed for stockpeople may be valuable in predicting their behaviour towards animals, providing useful information on the quality of HAR, and validated questionnaires have been used in the egg, dairy, pork and veal industries (reviewed by [82]).

Compared to tests aimed at assessing stockperson behaviour, attitude questionnaires devised for different farm species are easier to standardise and to deliver in a standardised manner and can be used to compare farming situations and studies. In addition, attitude questionnaires can be combined with direct assessments of animal welfare (e.g., [130]). However, it is worth noting that there is still a need for harmonization of attitudes assessments in research [99]. Attitudes can be assessed using either qualitative or quantitative methods or integrating them to obtain a better understanding of farmers’ attitudes towards animals and other aspects linked to animal welfare (e.g., [130]). A common approach used to investigate attitudes is to provide farmers with statements related to the object to be evaluated (e.g., dairy goats, adopting certain farm management practices, etc.), asking them to express their level of agreement on a Likert or semantic differential scale, and then inferring their attitudes by how they respond. Effects of interventions aimed at changing farmers’ attitudes can also be assessed [131]. 

Attitudes toward animals result to be linked to empathy toward animals [98]: however, empathy has been poorly investigated in stockpeople [92,97,110]. For example, based on previous research, Hanna et al. [97] created their own questionnaire to measure attitudes and empathy toward cows. Kielland et al. [110], in order to assess farmers’ empathy towards animals, developed and validated a photo-based pain assessment instrument depicting various conditions in cattle that could be associated with some degree of pain (PAI). As far as goats are concerned, Muri et al. [92] investigated attitudes and empathy in the goat industry involving a total of 260 dairy goat farmers. To our knowledge, this the first study assessing goat-oriented attitudes and empathy of stockpeople by means of questionnaires (web- and paper-based). In the study the attitude scale was based on the statements used by [13], while the empathy scale was an adaptation of the Animal Empathy Scale (AES) developed by [108]. Stockpeople behaviour was investigated in relation with work experience, other life experiences (e.g., being pet owner during childhood), demographic (e.g., age, gender) and educational level characteristics. This study reports that different dimensions of goat-oriented attitudes and empathy are associated with different demographic variables. It also suggests that the relationship between empathy and attitudes is complex and provides useful information for improving the methods for measuring animal oriented empathy in stockmanship [92].

Muri et al. [93] included a revised version of the previous questionnaire [92] during the development and testing of a welfare assessment protocol and stated that this tool has the potential to predict some welfare outcomes. For example, the authors found that, if farmers declared that they individually named most of their goats, the animals in those farms were calmer and less fearful [93]. During the validation of indicators of HAR, in [87,117] used a questionnaire originally developed for dairy cows to gather information about the attitude, empathy and behaviour of the stockperson towards goats, with researchers interviewing farmers directly on farm. Battini et al. [117] found that the predictive validity of the questionnaire is limited. It is argued that answers provided by farmers do not always correspond to their actual behaviour for a number of unclear reasons: the most likely is a conscious perception of social norms for which the farmer may respond dishonestly (i.e., it is unlikely that a farmer answers that beating or shouting at animals is a common practice). Presumably, anonymous questionnaires could provide more reliable answers.

## 6. Conclusions: Improving the Human–Goat Relationship

In several countries, animal farming has changed considerably and continues to change together with people’s attitudes towards farm animals and concern for their welfare and humane treatment [24,26]. Average farm size has been increasing, and farming is becoming more mechanized, resulting in the reduction of human–animal interactions and a lowered attention towards individual animals; this leads to growing difficulties in detecting abnormal behaviour and illnesses in animals [132] coupled with increasing animals’ fear of humans [133]. Conversely, animal welfare is now a relevant issue in many societies, and citizens’ and consumers’ attitudes towards farming, especially intensive farming, are changed, with a growing concern about animal welfare on farms [25]. 

Although animal welfare is a complex and multidimensional concept which allows for different definitions [134,135] and can be addressed differently depending on the actors involved (farmers, veterinarians, consumers), there is consensus among researchers, but also farmers, that HAR quality is relevant in all farming contexts in order to guarantee appropriate levels of animal welfare, and needs to be strongly encouraged and closely monitored, focusing on both the behaviour of the animals towards the stockpersons and the behaviour of the stockpersons towards the animals [18,136].

As noted by [137], farm animal welfare, and thus goat welfare, is not only a major concern for society and food production, but also represents an ethical issue. To improve goat welfare through positive human–goat interactions, it is essential to understand how goats communicate with humans, to gain knowledge on their behavioural and cognitive needs and capacities, and to promote and enhance this knowledge, especially in goat farmers, both at the theoretical and the practical level.

Species-specific studies on human–goat communication highlight that effective human–goat communication may take place by means of visual, tactile and auditory stimuli and, to a less extent, via olfactory and gustative stimuli, although further research may be useful to complement this knowledge. Spreading the information about this inter-specific communication means among stockpersons and people having contacts with goats may greatly help in improving the human–goat relationship and may also facilitate all handling and management procedures with animals. This will also be facilitated by spreading information about recent studies on farm animals’ socio-cognitive and affective capacities, which may have relevant implications for farm animal welfare and management [137,138,139,140,141]. Research on goats’ cognitive abilities suggests that goats have an understanding of their physical and social environment and a number of socio-cognitive abilities that affect their interactions with conspecifics and humans [47,137]. For example, goats can differentiate among conspecifics using visual and/or acoustic cues [70,142,143], and they can successfully learn from conspecifics or even from humans [60,62] and use cues provided by conspecifics or humans [49,63]. They can also differ in their anticipatory behaviour depending on an experimenter’s attentive state [62,63] and they are sensitive to human facial expressions [7]. This suggests that goats can adapt their behaviour based on the surrounding social environment. Therefore, it is extremely important that humans are aware of goats’ reactions and adaptive behaviour, and act in a way that can help goats to feel at ease.

Based on the evidence coming from comparative psychology, human–goat interactions and relationships could be improved by bridging scientific evidence on goats’ behaviour, cognition and emotions with practice: scientific knowledge needs to be shared with goat farmers in a functional and targeted way to enhance their capacity to correctly interpret goats’ intraspecific and human-directed behaviours and signals, thus favouring them in doing their job at best. Increasing farmers’ knowledge about goats’ behaviour, communication abilities, cognitive and emotional processes could help improving their housing and management conditions and could be used to evaluate the use and treatment of animals during production. Moreover, the enhancement of farmers’ knowledge and awareness that goats have cognitive abilities, experience positive and negative emotions and feel pain would be a useful prompt to perceive goats as individuals with internal states, and to develop more positive attitudes and a sense of commitment and responsibility for them [137]. The literature on human–animal relationship indicates that, among the many factors involved in determining attitudes towards other species, the belief in animal sentience or ‘‘mind’’ is a strong predictor of human attitudes toward different types of animal use [144,145,146].

Unfortunately, there is a lack of information about communication and interactions between humans and the “stem species” of domesticated goats—the wild goat *Capra aegagrus*. Therefore, it is not possible to compare the response behaviour of wild goats to humans with the behaviour of domestic goats. Further studies on behavioural interactions between humans and the still-existing wild ancestor of goats would be extremely useful to better understand and interpret the behavioural responses of domestic goats and to better understand the mechanisms and evolution of the human–goat relationship.

From the management point of view, there are several points where humans can have an active role to improve HAR. First of all, we have found five studies [17,54,80,84,85] highlighting that hand rearing of goat kids can have a positive effect on several aspects of the human–goat relationship. This apparently suggests to hand rear goat kids. However, there is a growing consumers’ hostility to the early separation of young animals from their mothers in the dairy industry. In cattle farming, early interactions with calves kept in cow–calf contact systems (e.g., assistance with suckling) is recommended to reduce future fear of humans [147]. The avoidance distance in cow-reared calves is shown to decrease with no differences compared to artificially reared calves in later ages (from calves to primiparous cows). Although there is a lack of specific studies in goats on this topic, it seems reasonable to think that raising animals with their mothers is not an obstacle to the establishment of a good HAR, but it does require specific knowledge from the farmer and, above all, humans have to keep in mind the importance of early contacts with kids, especially during their sensitive stages. In fact, frequent handling and frequent contacts between humans and goats have been found to improve the human–goat relationship [22]. Of course, it is also important to consider not only the frequency, but also the nature of the interactions. Gentling treatments, such as friendly talking, gentle touching, stroking and frequent hand feeding [19,48], may have a positive effect on HAR, whereas negative interactions, such as talking harshly, hitting and kicking the goats [87] have a negative impact. Furthermore, maintaining a low goat/stockperson ratio on farm, which implies more frequent opportunities for farmers to interact with their goats, also allows for the establishment of a more positive relationship [88]. 

Furthermore, as goats have well marked individual traits and temperaments, humans should learn to pay attention to individual animals, and genetic selection toward genotypes with a calm temperament, high docility and low timidity may also help to improve HAR.

A further useful way to improve the human–goat relationship would be to focus on the human side, understanding which farmers’ characteristics and behaviour allow to better combine good productivity, good practice and appropriate welfare standards. Farmers’ competence and technical skills, job motivations and satisfaction and attitudes combined with personality traits have been identified as job-related prerequisites for ensuring appropriate farm animal welfare standards [98,101]. These characteristics have been scarcely investigated in goat farmers so far (see [92,93,113]) and need to be further explored in future research. Sociodemographic variables (e.g., gender, cultural level) and personal characteristics (personality, empathy, and attitudes) have been shown to affect humans’ behaviour towards animals [93,95,101]. Gender differences have been well documented in the literature, with women showing higher levels of concern about animal suffering, holding more positive attitudes towards animals and being more engaged in animal protection and less prone to animal exploitation (see [103,148] for reviews). Since this has been confirmed also in goat farms [92], a greater involvement of women in goat farming should be welcome and fostered. Moreover, training programs aimed at promoting farmers’ development of positive feelings and empathic concern towards goats could be developed. Goat stockpersons’ personality (i.e., their pattern of thoughts, feelings and behaviours) has not been investigated; however, personality traits affect human behavioural responses towards both people and animals and relate to both animal welfare and productivity (e.g., [96]). Goat farmers’ attitudes deserve investigation, since attitudes are key determinants of human behaviour towards people and animals, and their influence on interactions with farm animals, productivity and management has been shown for different farmed species (e.g., pigs: [149]; cattle: [96,150,151]). It is worth noting, however, that stockpersons’ attitudes toward animals are not just limited to the direct interactions with them, such as handling, but also affect attention to details, readiness to solve problems, decisions in management and housing decisions; thus, understanding the role of attitudes in goat farming would be particularly useful. Given that attitudes are learnt and may change over time because of new information, new experiences and newly acquired knowledge [152], appropriate training based on specific experiences and knowledge acquisition could have a positive effect in changing farmers’ attitudes, reducing negative behaviours and poor relationships. According to [15], to ensure that training programmes are well targeted for farmers, they should be adapted to situations, needs and level of knowledge that humans have already acquired.

In practice, while there is a fair number of validated measures for assessing the human–goat relationship, many of these are recorded by unfamiliar assessors, and they are not designed for a feasible self-assessment from farmers or owners. However, knowing the meaning of some of the goats’ reactions towards humans, e.g., avoidance and flight reaction or voluntary approach to a familiar person, can help to identify possible issues. In addition, the behaviour of goats in the presence of unfamiliar people (e.g., visitors, children from schools) can also be observed to see how goats perceive the human presence and if the animals need to be more accustomed to people.

A good human–goat relationship implies a number of work-related aspects including competence and technical skills, which are important also in implementing farmers’ job satisfaction. Job satisfaction is associated with more positive attitudes and emotions, and with greater farmers’ self-efficacy and wellbeing [153,154,155]. Farmers’ job satisfaction and wellbeing can be viewed as prerequisites for optimal stockmanship and should be investigated and implemented to improve goat welfare. As claimed by [155], animal welfare is a multi-faceted issue with scientific, ethical, economic and political dimensions, and it requires a multidisciplinary approach to broaden the understanding of the human–goat relationships aimed at improving the lives of both humans and animals.

## Figures and Tables

**Table 1:** Effective intentional communication signals between goats and humans, and vice versa.

Signal Category ^1^	Behaviour	Emitter	Receiver	Meaning/Goal	Ref.
A	Acoustic signals at different tones	Human	Goat	Training to shape discrimination	[52]
A	Clicker sound previously associated with food reward	Human	Goat	Training to wear a halter	[53]
A	Loud vocalizations	Human	Pregnant goat	Negative handling treatment	[23]
A	Speaking	Human	Goat kids	Positive handling treatment	[54]
A	Speaking in a soft voice	Human	Pregnant goat	Positive handling treatment	[23]
A	Speaking in a soft voice	Human	Goat	Inviting goats to approach + positive handling treatment	[55]
A	Vocal call “Come here”	Human	Goat	Call for goat’s attention	[36]
A	Vocal call “Come here” or “Come on, honey”	Human	Goat kids	Call for goat’s attention	[50]
G	Licking	Goat	Human	Positive feelings, search for contact	[56]
O	Smelling	Goat	Human	Positive feelings, search for contact	[56]
T	Biting and pulling human’s clothes	Goat	Human	Negative feelings, discomfort	[56]
T	Contact alternation (frequency and latency)	Goat	Human	Asking for help to solve problem	[48]
T	Establishing physical contact (rubbing, nosing, pawing a hand or leg or jumping up)	Goat	Human	Asking for help to solve problem	[57]
T	Physical contact (latency and duration)	Goat	Human	Asking for help to solve problem	[58]
T	Pushing human’s arm and hands with head/horns	Goat	Human	Negative feelings, discomfort	[56]
T	Rubbing the head, placing it on human’s lap	Goat	Human	Positive feelings, search for contact	[56]
T	Brushing head and back	Human	Goat	Inducing changes of emotional state	[59]
T	Massage	Human	Goat	Promoting goats’ relaxation, improvement of HAR	[56]
T	Petting, scratching, stroking	Human	Goat	Positive handling treatment	[55]
T	Petting, stroking and scratching	Human	Pregnant goat	Positive handling treatment	[23]
T	Stroking	Human	Goat kids	Positive handling treatment	[54]
T	Stroking	Human	Goat	Positive handling treatment	[19]
T	Touching, stroking and brushing	Human	Goat	Establishing contact with goats	[46]
T/G	Chewing (contact of goat’s mouth with humans)	Goat	Human	Refusing to wear a halter	[53]
T/G	Nibbling	Goat	Human	Positive feelings, search for contact	[56]
T/G	Nibbling human clothes	Goat	Human	Two hypotheses: search for social contact or replacement behaviour in a poorly enriched environment?	[50]
V	Approaching	Goat	Human	Establishing contact with humans	[55]
V	Establishing visual contact	Goat	Human	Asking for help to solve problem	[57]
V	Gaze alternation (frequency and latency)	Goat	Human	Asking for help to solve problem	[48]
V	Gazing	Goat	Human	Searching for cues on hidden food	[60]
V	Moving away from the trainer	Goat	Human	Refusing to wear a halter	[53]
V	Moving toward the trainer	Goat	Human	Establishing contact with the trainer	[53]
V	Standing in front of the trainer	Goat	Human	Establishing contact with the trainer	[53]
V	Turning (90°) of goat’s neck/head	Goat	Human	Refusing to wear a halter	[53]
V	Turning head and directing gaze away from the milk bottle	Goat kids	Human	Not interested in drinking milk	[50]
V	Body orientation	Human	Goat	Stimulating approach behaviour	[36]
V	Facial expressions	Human	Goat	Stimulating approach and interaction	[7]
V	Gazing	Human	Goat	Indicating a given direction	[61]
V	Head and body orientation	Human	Goat	Providing for cues on hidden food	[62]
V	Offering food	Human	Goat	Inviting goats to approach	[50]
V	Offering food (twigs)	Human	Goat	Inviting goats to approach	[46]
V	Open vs. closed eyes	Human	Goat	Stimulating approach behaviour	[36]
V	Pointing the arm	Human	Goat	Providing cues on hidden food	[49]
V	Pointing the arm	Human	Goat	Providing cues on hidden food	[63,64]
V	Slow arm and hand movements	Human	Goat	Inviting goats to approach	[55]
V	Touching object	Human	Goat	Providing cues on hidden food	[63]
V	Touching object and moving the arm	Human	Goat	Providing cues on hidden food	[49]
V/A	Shacking a food container	Human	Goat	Attract goats’ attention	[65]

^1^ V: Visual signals; A: Acoustic signals; O: Olfactory signals; T: Tactile signals; G: Gustatory signals.

**Table 2:** Factors affecting the human–goat relationship.

Factor Type ^1^	Factor	Effect on HAR	Ref.
E	Human rearing of goat kids	>time of kids in proximity with humans in goat-human encounter test <latency of kids to approach humans in goat-human encounter test <kids’ flight distance in goat-human encounter test	[80]
E	Human rearing of goat kids	<kids’ avoidance distance in AD ^2^ test	[84]
E	Human rearing of goat kids	>confidence of kids with humans >ease of management in adulthood	[85]
E	Human rearing of goat kids	<flight distance in encounter and choice test >time in proximity with humans in encounter and choice test	[54]
E	Human rearing of goat kids	<latency of adult goats to approach human in encounter test in the home pen >time of adult goats in proximity of humans in encounter test in the home pen	[17]
E	Frequent contacts with visitors (in zoo)	>human-directed behaviour in an impossible task paradigm	[57]
E	Frequent contact with humans (entering the goat pen twice/day and walking calmly among the goats for 20 min)	<flight speed of feral rangeland goats in a flight response test	[22]
E	Farming system (intensive vs. semi-extensive)	<latency to the first contact in intensive farms	[86]
E	Gentling treatment (friendly talking, gentle touching, stroking and hand feeding twice daily, over two weeks)	Performing of alternation of gaze and contact towards the human being in an unsolvable task paradigm	[48]
E	Gentling treatment (stroking goats’ back and neck with eye contact for 10 min for 24 days)	Quicker approach to the experimenter in latency test faster habituation to the presence of the experimenter	[19]
E	Proportion of negative interactions during milking (e.g., talking harshly, hitting and kicking the goats)	>avoidance behaviour in goats; <approach behaviour in goats	[87]
E	Small farm size, low goats/stockperson ratio	>% of acceptance and contact with human in AD ^2^ test	[88]
E	Presence of environmental enrichments (i.e., tractor tyres; heap of compacted earth)	>distance from the human experimenter in a handling test	[81]
I	Temperament	>latency to proximity and latency to contact in different test situations in “timid” goats	[17,80]
I	Breed	Boer vs. Tswana, Nguni and Xhosa lob-eared genotype: >flight times during handling; <flight speed during handling; <vocalization score during handling; <crush score (CS) ^3^ during handling	[89,90]
I	Breed	Saanen and Murciano-Granadina easier to handle than more rustic breeds (hypothesis)	[91]
I	Low social rank	>proximity to a stationary human; <time to get used to stress due to handling and restraint	[81]
I	High social rank	>distance from a stationary human; >time to get used to stress due to handling and restraint	[81]
H	Considering goats pleasant animals	>possibility of pain recognition in goats	[92]
H	Higher empathy	>positive attitudes in relation to goats and working with them	[92]
H	Being raised on a farm	<human consideration of the goat as a pleasant animal to work with, entertaining and intelligent	[92]
H	Stockperson’s gender	in women >ability to interpret and understand goats’ experience and find easier to work with goats	[92]
H	Belief in the importance of positive contact with goats (i.e., stroking)	>goats’ willingness to be touched	[93]

^1^ E = environmental; I = individual; H = human. ^2^ AD = avoidance distance. ^3^ CS = crush score: behaviour of an animal assessed when put into a crush, using a 1 (calm) to 5 (combative) scale.

**Table 3:** Behavioural tests for evaluating the quality of human–goat relationship.

Category	Assessor Behaviour	Assessor	Animal Category	Test Context	Social Context	Procedure	Variables	Validity ^1^	Ref.
Stationary human	Motionless	Not specified	Adult	Test environment	Isolation	Goat restraint in a starting zone for 45 s and released in the arena with an assessor standing	Latency of proximity with the human, duration in proximity (within 2 m), sections crossed, mean distance from the humans	Y	[80]
Stationary human	Motionless	Unfamiliar	Adult	Test environment	Audience	Goat placed 5 min in the arena, peers behind a fence, then assessor enters and stays still for 5 min. Heart rate recorded by telemetry.	Duration in contact with the human, number of vocalisations, heart rate.	Y	[115]
Stationary human (1); Moving human (2)	(1) seated human standing still, but moving the hand; (2) human approaching	Familiar	Kids	Test environment	Isolation	Two phases. Seated human: goat left alone in arena for 1 min, assessor enters and stands still for 1.5 min, stroking the kid if approaching. Moving human: the assessor approaches and tries to pet kid for 1.5 min	Duration in proximity (<2 m), in contact with the human. Vocalisation, sections crossed	Y	[54]
Stationary human; Moving human	(1) and (2) motionless; (3) human approaching	Unfamiliar	Adult/Kids	Home environment, indoor	Group/Isolation	Three phases. (1) assessor enters pen and stands still, (2) assessor moves back and forth along the front fence, (3) assessor tries to touch goats	Latencies to approach the human (<1 m) and to make contact, duration in proximity (stationary or moving)	Y	[17,116]
Moving human	Human approaching	Unfamiliar	Adult	Test environment	Isolation	Goat placed in a circular runway, assessor walks (0.5 steps/s) behind it for 3.5 min. Blood sampling taken 3 days before the test, immediately after and 3 days after	Mean flight distance, following, approach, avoidance, vocalisation, human contact, urination (plasma cortisol)	Y	[80]
Handling	Handling	Familiar	Adult	Home environment, indoor	Group	Goats milked twice daily for 21 days by two persons. Then, the same persons score each goats behaviour	Seven behavioural scales: excitable, tense, watchful, apprehensive, confident, friendly to humans, fearful of humans. Milk ejection.	Y	[17]
Stationary human	Motionless	Unfamiliar	Adult	Home environment, indoor	Group	Assessor enters the pen and walks to a pre-determined spot, marking a 1.5-radius semi-circumference and starts the stopwatch. Assessor stands motionless for 5 min, back against the wall	Latency to the first contact performed by the first goat, percentage of goats that nuzzled or touched any part of the assessor (continuously recorded and at 1 min-scan), percentage of goats that entered the semi-circumference around the assessor, at 1 min-scan	Y	[117]
Moving human	Human approaching	Unfamiliar	Adult	Home environment, indoor	Group	The assessor enters the pen and stands in front of a goat (randomly chosen) at a distance of 300 cm, then starts moving slowly towards the animal at a speed of one step/s, 60 cm/step and the arm lifted with an inclination of 45°, the hand palm directed downwards, without looking into the animal’s eyes, but looking at the muzzle. When the goat shows the first avoidance reaction (moving back-wards, turning or shaking its head), the assessor recorded the distance between the hand and the muzzle of the animal, with a resolution of 10 cm. If the animal can be touched by the assessor, the distance is 0, and this is also defined as “contact”.	Mean avoidance distance (cm) of the goats tested in the pen, percentage of goats that can be touched by the assessor during the AD test	n.t.	[114]
Moving human	Human approaching	Unfamiliar	Adult	Home environment, indoor	Group	Same procedure as [114] but with a starting distance of 200 cm. If the animal can be touched by the assessor but immediately withdraws, this is recorded as “contact”; if, after the contact, the animal accepts gently stroking of the head for at least 3 s, this is recorded as “acceptance”.	Mean avoidance distance (cm) of the goats tested in the pen, percentage of goats that nuzzle or touch the hand of assessor during the AD test, percentage of goats that accept gently stroking of the head by the assessor for at least 3 sec during the AD test, percentage of goats tested	Y	[117]
Stationary human	Motionless	Unfamiliar	Adult	Home environment, indoor	Group	Assessor enters the pen and walks to a pre-determined spot, possibly in the middle of the long side of the pen. Then starts the stopwatch and stands motionless for 5 min, back against the wall	Latency to the first contact performed by the first goat	Y	[86,91,117,118]
Moving human	Human approaching and/or calling	Familiar	Adult	Home environment, outdoor	Group	The farmer approaches the goats in the usual manner. The assessor (out of sight of the animals) records the reaction of goats toward the farmer.	Three possible reactions of goats are recorded: avoidance, contact, approach	n.t.	[119]
Moving human	Human approaching and/or calling	Familiar	Adult	Home environment, outdoor	Group	The closest distance (m) of approach the group, before a flight response is evoked, is recorded. If an animal stands motionless, this is recorded as 0 m. Animals that voluntary approach the farmer and/or interact (sniffing or touching) are also recorded.	Mean avoidance distance (cm), percentage of animals voluntary seeking for human contacts	n.t.	[120]
Handling	Handling	Familiar	Adult	Home environment, indoor	Group	Unaware of being tested, the stockperson approaches and marks individual pre-selected goats on the head with a marking crayon, while an assessor evaluate his/her behavioural style, as well as the goats’ behavioural responses during the procedure	Behavioural responses registered on five-point rating scales (1 = positive interactions; 5 = negative interactions)	Y	[93]
Stationary human	Human standing still, moving the hand	Unfamiliar	Adult	Home environment, indoor	Group	Chin contact test—The assessor stands in front of each goat, reaches out an arm with the palm pointing upwards, and gently moves the hand towards the goat’s chin.	The goat’s response to the hand is registered on a three-point scale: full acceptance, brief touch, full avoidance	Y	[93]
Moving human	Human approaching	Unfamiliar/familiar	Adult	Test environment	Group	3-min human approach test conducted after first- and seventh-handling experience of goats. Three main categories of reactions: (1) spatial (close, middle, far), (2) orientation (facing vs. turned-away), (3) structural (lie, stand, and nutritive and non-nutritive oral behaviours).	Percentage of duration of behaviour outcomes to create an approach index (AI): great approach (≥75% quartile), moderate approach (25% to 75% quartiles), least approach (≤25%)	n.t.	[121]
Moving human	Human walking along the feeding alley	Unfamiliar	Adult	Home environment, indoor	Group	Avoidance test at the feeding place—The assessor walks on the feeding alley with 0.5 steps/s, at a distance of about 80 cm parallel to the feed barrier, assessing the reaction of feeding goats as the assessor passes by,	Percentage of animals still feeding when the assessor passes by	Y	[87]
Moving human	Human approaching	Unfamiliar	Adult	Home environment, indoor	Group	Avoidance distance test at the feeding place—From a distance of 200 cm, the assessor approaches individual animals than stand at the feeding place, constantly walking (speed of 0.5/s, steps of about 30–40 cm) with one arm 45° in front of the body, fingertips pointing to the ground and back of the hand towards the goat, until the goat withdrew or until touching. In case the goat can be touched but withdraws within 2 s an avoidance distance of 1 cm is assigned. Only when a goat accepts being stroked for more than 2 s an avoidance distance of 0 cm is assigned	Median value of avoidance distance at the feeding place, percentage of animals possible to stroke, percentage of animals with an avoidance distance greater than 1 m	Y	[87]
Stationary human	Motionless	Unfamiliar	Adult	Home environment, indoor	Group	Approach test in the pen—The assessor enters the pen and after a 30–34 s pause walks to a pre-decided testing place in the pen and marks three positions in a radius of 3 m. Then, the assessor stands 15 min motionless with the back to a wall.	Absolute number of goats into physical contact with the assessor, latency of the first animal touching the assessor (1-min scan), average number of goats within the 3 m radius (1-min scan), proportion of goats within 0.5 m to the assessor during the first 5 min	N	[87]
Moving human	Human approaching	Unfamiliar	Adult	Home environment, indoor	Group	Avoidance test in the pen—Two successive phases. Phase 1: the assessor walks for 1–2 min through the pen and observes the distance of the goats being closest to him/her. Phase 2: after leaving the pen for at least 2 min, the assessor re-enters the pen and approaches single animals, trying to touch them	Phase 1: estimation of the average distance from the group over the whole time. Phase 2: percentage of animals that can be touched	Y	[87]
Moving human	Human approaching	Familiar	Adult	Test environment	Group	Starting from a distance of 20 m, the assessor approaches the goats at a slow walking speed (1.5 m/s). When the flight response is induced, the assessor stops still after all the goats have run past.	Distance that the assessor approaches the group of goats at the time that all the goats run past, average speed at which the goats run past and away from the assessor	N	[22]
Stationary human	Motionless	Unfamiliar	Adult	Test environment	Isolation	The assessor keeps the eyes on the goat without moving the face or body for 5 min.	Behaviours: gazing, proximity (within 50 cm), contacting (at a distance of 1 to 10 cm)	n.t.	[58]
Moving human	Human approaching	Unfamiliar	Adult	Test environment	Isolation	The assessor approaches a goat leashed (1-m rope) on the side of the paddock, walking obliquely at a pace of 1 step/sec. If the goat remains stationary within 1.5 m, the assessor slowly moves the hand close to the face of the goat. If the goat does not escape and tries to smell the hand, the assessor tries to touch the goat’s neck.	Scores (1 to 4): (1) goat moves away from the assessor (>1.5 m range), (2) goat stands still when the assessor is within 1.5 m range, (3) goat sniffs the assessor’s hand, (4) the assessor touches goat’s neck	n.t.	[58]
Stationary human	Motionless	Unfamiliar	Adult	Home environment, indoor	Group	The assessor moves to approximately the middle of the pen and begins timing the latency for each animal to approach within 60 cm. This measurement is capped at 10 min regardless of whether or not the animal approaches.	Latency to approach	n.t.	[19]

^1^ Y = yes; N = no; n.t. = not tested.

## Data Availability

Not applicable.
